# Protein microarray for complex apoptosis monitoring of dysplastic oral keratinocytes in experimental photodynamic therapy

**DOI:** 10.1186/0717-6287-47-33

**Published:** 2014-07-18

**Authors:** Clara Matei, Mircea Tampa, Constantin Caruntu, Rodica-Mariana Ion, Simona-Roxana Georgescu, Georgiana Roxana Dumitrascu, Carolina Constantin, Monica Neagu

**Affiliations:** Dermatology Department, “Carol Davila” University of Medicine and Pharmacy, 8, Bulevardul Eroii Sanitari, Bucharest, 050474 Romania; Department of Physiology, “Carol Davila” University of Medicine and Pharmacy, Bulevardul Eroii Sanitari, Bucharest, 050474 Romania; The National Institute for Research & Development in Chemistry and Petrochemistry, 202, Splaiul Independentei, Bucharest, 060021 Romania; Immunobiology Laboratory, “Victor Babes” National Institute of Pathology, 99-101, Splaiul Independentei, Bucharest, 050096 Romania

**Keywords:** Aluminum di-sulphonated phthalocyanine, DOK cells, Photodynamic therapy, Protein microarray

## Abstract

**Background:**

Photodynamic therapy is an alternative treatment of muco-cutaneous tumors that uses a light source able to photoactivate a chemical compound that acts as a photosensitizer. The phthalocyanines append to a wide chemical class that encompasses a large range of compounds; out of them aluminium-substituted disulphonated phthalocyanine possesses a good photosensitizing potential.

**Results:**

The destructive effects of PDT with aluminium-substituted disulphonated phthalocyanine are achieved by induction of apoptosis in tumoral cells as assessed by flow cytometry analysis. Using protein microarray we evaluate the possible molecular pathways by which photodynamic therapy activates apoptosis in dysplastic oral keratinocytes cells, leading to the tumoral cells destruction. Among assessed analytes, Bcl-2, P70S6K kinase, Raf-1 and Bad proteins represent the apoptosis related biomolecules that showed expression variations with the greatest amplitude.

**Conclusions:**

Up to date, the intimate molecular apoptotic mechanisms activated by photodynamic therapy with this type of phthalocyanine in dysplastic human oral keratinocytes are not completely elucidated. With protein microarray as high-throughput proteomic approach a better understanding of the manner in which photodynamic therapy leads to tumoral cell destruction can be obtained, by depicting apoptotic molecules that can be potentially triggered in future anti-tumoral therapies.

## Background

Photodynamic therapy (PDT) is a therapeutical approach directed towards destruction of tumoral cells, with increasing use in various medical fields such as urology, gastroenterology, pneumology and ophthalmology. The anti-tumoral effect of PDT is widely used in dermatology, in the treatment of several muco-cutaneous tumors, such as basal and squamous cell carcinoma, Bowen disease, leucoplakia and oral dysplasia [1, 2].

**Figure 1:**
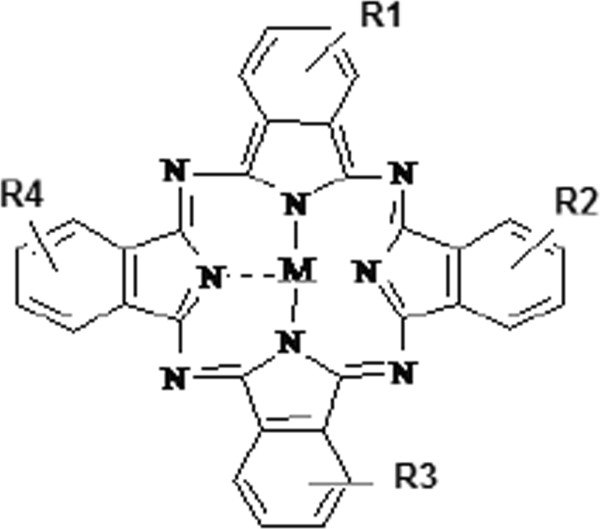
**The structure of metallo-sulphonated phthalocyanines (M = metal; R = SO3-/H).**

PDT uses an adequate light source able to photoactivate a chemical compound that acts like a photosensitiser; various photosensitizers are recently the subject of intense research worldwide, mainly porphyrin precursors and derivatives such as the phthalocyanines [3]. The latest are chemical structures sharing a four-ring pyrrol-like aromatic structure similar to a certain extent to that of the porphyrins; central inclusion of a metal ion such as Al, Zn, In or Ag modifies the photodynamic properties of the phthalocyanines (Figure 1). The central metal ion modulates the photodynamic properties by acting through the alteration of the electron density in the core of the compound, increasing π-electron delocalization and consequently changing the absorbance spectrum of the substance [4].

Aluminium di-sulphonated phthalocyanine (AlS2Pc) is a metallo-substituted phthalocyanine with increased solubility, due to the sulphonated substituents from the macrocycle periphery. We have previously assessed the photodynamic properties of AlS2Pc and established its dark-toxicity pattern and its optimum activation upon PDT [5].

PDT destroys tumoral cells mainly by induction of apoptosis, the programmed cell death, a complex process consisting in the activation of multiple intracellular signaling pathways culminating with caspase cascade stimulation that leads to nuclear dissolution, DNA fragmentation and lysis of various protein substrates vital to normal cell physiology. Apoptosis is the result of a genetic encoded program activated by various stimuli. Induction of apoptosis in the tumoral cells in a selective manner, without alteration of the surrounding normal cells is a desired effect that PDT relies upon and this selective process is achieved by accumulation of the photosensitizer preferentially in the tumoral cells. After illumination of the cells containing the photosensitiser with light of an appropriate wavelength, the energy provided by the light source is absorbed by the photosensitiser and consequently discharged in the medium surrounding the cell, leading to photodynamic reactions that finally activate apoptosis [1]. The particular manner in which apoptosis is activated depends of the cell type and the photosensitiser used in the process. The mechanistic aspects employed by PDT using Zn-substituted tri-sulphonated phthalocyanine in DOK cells have been previously described by us [6].

**Figure 2:**
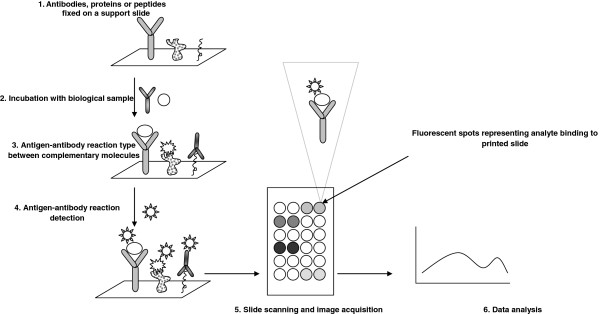
**Protein microarray technology principle.**

Protein microarray analysis has started to be increasingly used in various biomedical applications, but few papers are published using this technology for evaluating the complex apoptosis network triggered by PDT therapy. The basic principle and a schematic workflow for protein microarray technique are shown in Figure 2. Recently published 5-Aminolevulinic acid (ALA) used to photosensitize mouse cerebral cortex took advantage of proteomic antibody microarrays. Using this method 112 proteins were detected with supposed epigenetic regulation potential. Among these proteins, the PDT using ALA precursor of porphyrin induced histone H3 d**i**methylation as well as up-regulation of protein Kaiso, a DNA methyl-binding protein, the overall effect being the suppression of the transcriptional activity in photosensitized cells. This recent study showed that protein microarray can furnish high-throughput results further to be used in therapeutical approaches. Moreover, this study showed that new therapeutical targets can be revealed upon assessment of epigenetic markers [7].

Publications released at the beginning of 2000 focus only on gene microarray technologies used for apoptosis quantification post-PDT [8, 9], later on transcriptomic deregulations [10, 11] and, only in the last years several publications focused on protein microarray evaluation, mainly due to the early stage in which protein-microarray technology was 10 years ago.

Although a few studies presented the overall pattern of apoptosis induction in PDT using aluminum di-sulphonated phthalocyanine [12–14], until now, as to the best of our knowledge, there are no studies directed towards clarifying the molecular mechanism involved in the induction of apoptotic cell death following PDT using AlS_2_Pc. Such studies could reveal the intimate mechanisms of apoptosis induction, therefore evaluating in depth this process other biomolecules can be revealed as potential targets for enhancing the PDT-apoptosis effect. For that reason, the presented results aimed to establish the interrelation of apoptotic proteins that are activated by PDT using AlS_2_Pc in DOK cells.

## Results and discussion

### Apoptosis

**Figure 3:**
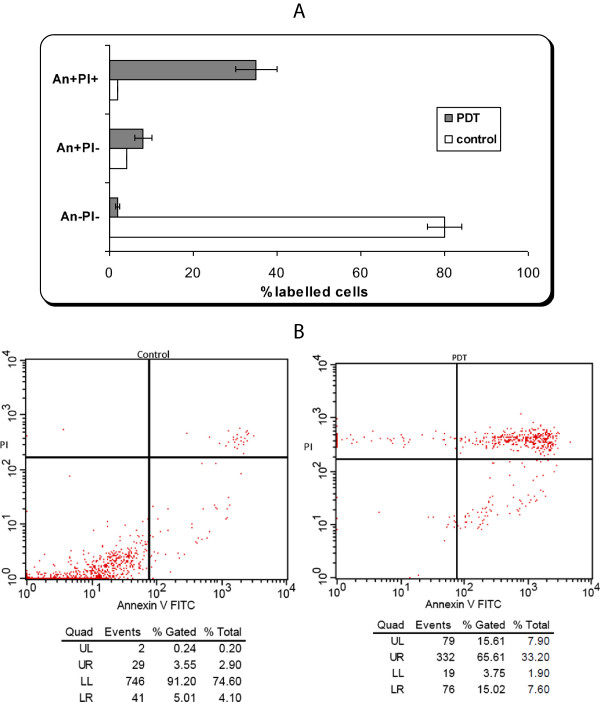
**Apoptosis evaluation by flow-cytometry of DOK cell line subjected to PDT. A)** Apoptosis evaluation for triplicates repeated in four identical experiments. Annexin V-FITC and PI (red) quantified early, late apoptosis and necrosis: An-Pi- viable cells; An + Pi- early apoptotic cells; An + Pi + late apoptotic/necrotic cells. Cell samples are from the same batches that were subjected post-irradiation to Protein microarray analysis. **B)** Example of flow cytometry registration of un-loaded (control) and loaded cells with AlS_2_Pc and subjected to PDT.

In order to prove that the apoptosis mechanisms were induced upon PDT with AlS_2_Pc we have used flow cytometry evaluation of irradiated non-loaded cells (control) and loaded cells (PDT). From the same cell batches that were subjected to *Protein microarray* evaluation, at 1.5 hours after irradiation protocol, photosensitizer loaded cells showed a percentage of around 15% cells stained Ann positive and PI negative (early apoptotic events) and nearly 40% cells staining double positive for PI and Ann (late apoptotic events) (Figure 3). The finding that upon PDT mainly late apoptotic events are induced is consistent with previous data [15]. Moreover, the fact that we have found that even cellular membrane is affected upon experimental PDT, as assessed by Ann staining, can either depict the fact that there are still cells that will enter the late apoptotic event and/or as previously published [16]. These types of phthalocyanines can reside in early endosomes thus hindering *per se* the membrane architecture, hence the positivity of annexin staining.

### Protein microarray

**Figure 4:**
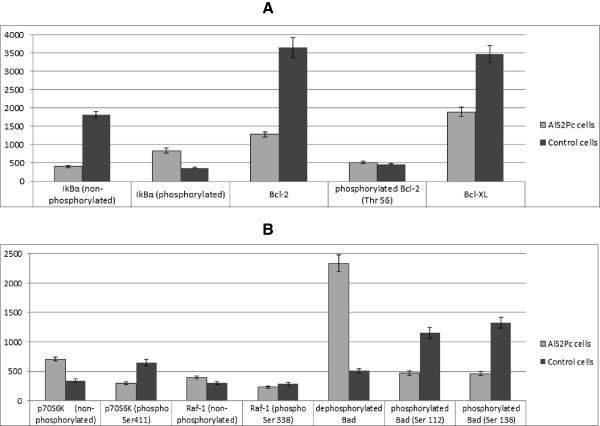
**Results of protein microarray analysis of the main proteins involved in apoptosis in DOK cells following PDT using AlS2Pc; mean values and standard deviations are reported, measured in arbitrary fluorescent units.** Control cells: un-loaded irradiated DOK cells; AlS2Pc cells: loaded irradiated DOK cells.

We have analyzed the apoptosis related biomolecules that showed the variations with the greatest amplitude (Figure 4). Anti-apoptotic proteins *Bcl-2* ("B-cell lymphoma 2") and Bcl-XL ("B-cell lymphoma 2 extra large") block apoptosis in usual conditions; their increase inhibits apoptosis, while their variation in the opposite direction facilitates programmed cell death [17]. This modulator role of Bcl-2 proteins in cell apoptosis triggered by PDT is still controversial. The overexpression of Bcl-2 could either be efficient or abstaining in tumor cells killing upon experimental PDT, depending on cell type and/or photosensitizer nature [18].

We have observed a decrease by 64.87% of Bcl-2 level (p < 0.001), accompanied by a decrease by 45.5% in the level of Bcl-XL proteins in DOK cells treated with AlS2Pc and subjected to PDT. Bcl-2 activity is regulated not only by alteration in the level of protein expression, but also by various chemical structure alteration carried by several enzymes. Bodur et al. have shown in a recent study [19] that Bcl-2 can be phosphorylated by IKK enzyme (IkBα-kinase) at a threonine residue located in the 56^th^ position of the protean chain, leading to a conformational modification expressed by a decrease in the antiapoptotic action of Bcl-2, consequently promoting apoptosis. Our experimental data shows an increase by 11.62% (p = 0.008) of phospho-Thr 56 Bcl-2 in DOK cells incubated with AlS2Pc after PDT, as compared to the control cells.

This change in phosphorylated form of *Bcl-2* is connected to the increase in the activity of IKK; usual substrate of IKK is protein IkBα, the inhibitor of nuclear factor *kb* (NFkB); under certain conditions, IKK phosphorylates IkBα [20]. The activation of IKK was revealed in the experiment we have carried on by the finding of 2.287 folds increase of phosphorylated form of IkBα, altogether with a correspondent decrease of 4.54 folds in the level of dephosphorylated form of IkBα. In a somewhat related PDT study applied to glioblastoma cells was assessed by using protein microarray early molecular events in cells subjected to photosensitizing. Sub-lethal experimental PDT approach was used in the prior published study, where time dependent-, subtle molecular proteomic events were depicted. The entire group of 224 proteins expressed after this almost non-lethal PDT were studied. Antibody microarrays showed that in the first hour of PDT treatment several proteins, such as, protein kinase Raf, adhesion-related kinases FAK and Pyk2, and microtubule-associated protein tau are phosphorylated. In this study Bcl-xL was found over expressed, while caspase 9 down-regulated an overall anti-apoptotic response [21]. The differences in the actual results we are presenting compared to the previous reported PDT, besides the different type of cell and PDT photosensitizer resides in the dose type that is a sub-lethal one in the previous reported study [21] while in ours we have used lethal doses.

*P70S6K kinase* is a factor with important roles in protein synthesis and cell growth. Besides these functions, p70S6K is involved in apoptosis regulation by phosphorylation of apoptosis enhancer BH3-only protein Bad ("Bcl-2-associated death promoter"). Bad phosphorylation leads to its sequestration in the cytosol by 14-3-3 proteins, explaining p70S6K inhibition of apoptosis [22]. The active form of the p70S6K enzyme is the phosphorylated state of the protein. Serine residue in 411 position is of paramount importance in this process, because in the absence of its phosphorylation p70S6K cannot be activated [23]. We have observed an important increase of more than two folds of the p70S6K dephosphorylated form. Alongside, there was a decrease by 54.2% (p value <0.001) in the level of p70S6K phosphorylated at Ser411, demonstrating a decline of the p70S6K active form; whilst p70S6K inhibits apoptosis by Bad phosphorylation. Thus, we can decode the reduction of activated p70S6K level as a pro-apoptotic effect of AlS2Pc-PDT in DOK cells.

*Raf-1* serine-threonine kinase ("cellular-rapidly accelerated fibrosarcoma", c-Raf) is a protein that plays multiple key roles in various intracellular signaling processes, as well as in controlling cell growth and proliferation. Raf-1 is also involved in apoptosis regulation, by Bad phosphorylation, with subsequent blocking of Bad protein function [24]. Hence, Raf-1 kinase must be previously activated by phosphorylation in order to exert its Bad modulating activity [25]. We have observed a decrease by 20.45% of the phosphorylated form of Raf-1 at Ser338, accompanied by a raise by 31.05% of the non-phosphorylated Raf-1 level (p < 0.001). Taking into consideration that Raf-1 activation promotes cell survival by phosphorylation and inactivation of Bad protein**,** the observed alterations in the Raf-1 kinase level forms represent a proapoptotic effect of AlS2Pc-PDT in cultured dysplastic oral keratinocytes.

*Bad* is a regulating protein able to dissociate proapoptotic-antiapoptotic protein dimers, releasing pro-apoptotic proteins from inhibition exerted by the anti-apoptotic counterparts Bcl-2 and Bcl-XL. The active pro-apoptotic form of Bad is the dephosphorylated form, thus Bad phosphorylation at Ser112 and 136 leads to its association with 14-3-3 proteins, blocking the Bad activity [22, 26]. We have acknowledged an increase of 4.59 folds of dephosphorylated Bad, accompanied by a reduction by 59.4% of Bad phosphorylated at Ser112 and 65.3% of Bad phosphorylated at Ser136, data that consequently proves the involvement of Bad protein in the induction of apoptosis in DOK cells following AlS2Pc-PDT. These observations are also consistent with our previous data regarding the activation status of Bad [5], as the decrease of Bad phosphorylation can be attributed to the lack of phosphorylation (and therefore activation) of p70S6 kinase and Raf-1 kinase.

**Figure 5:**
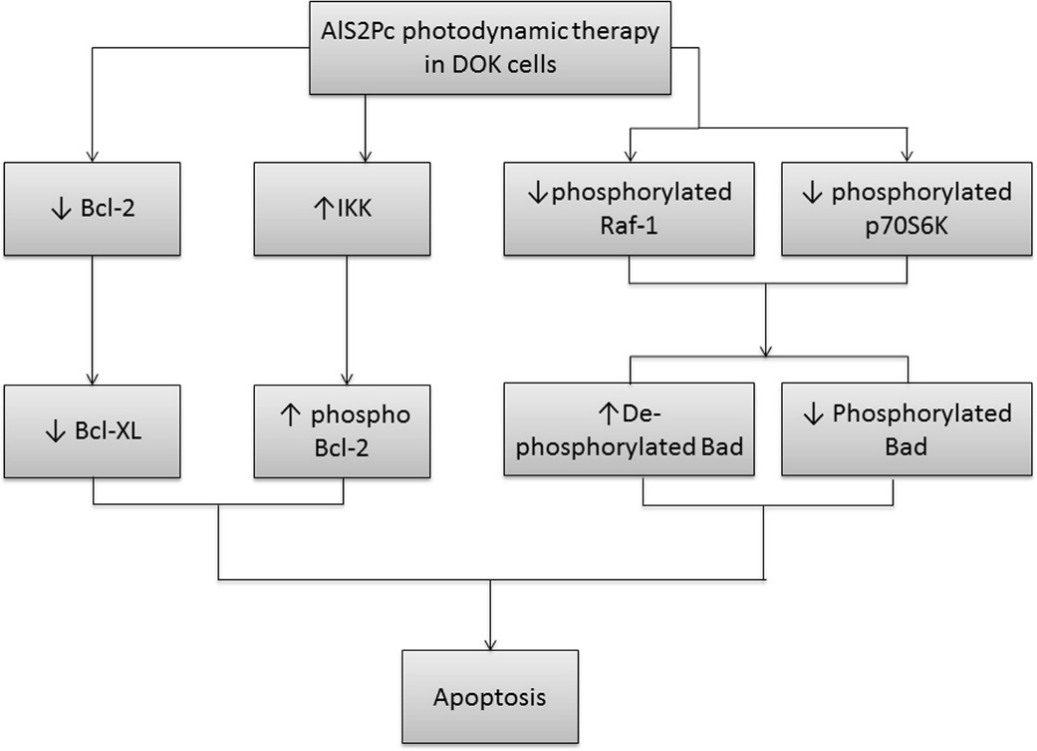
**Molecular mechanisms activated by photodynamic therapy using AlS2Pc in DOK cell line – delineative representation resulted from protein microarray analysis.**

Thus photodynamic therapy using aluminum disulphonated phthalocyanine leads to induction of apoptosis in cultured human dysplastic oral keratinocytes DOK following a distinctive molecular pathway (Figure 5). The changes in the level of the main proteins involved in apoptosis are consistent with apoptosis activation *via* intrinsic mitochondrial apoptotic pathway.

Similar to our experimental approach, but using reverse phase protein microarray in a human urothelial cell lines model, several apoptotic proteins were found altered upon PDT. This study has reported that caspase-3 and -9, HIF-1alpha, Bcl2, Cox2 and the phosphorylated AKT were enhanced upon PDT with ALA. The authors point out that, when the cells display a higher differentiation phenotype the apoptotic response is increased, while lower differentiated cells can have truncated apoptosis pathways hence their resistance to therapy [27]. This sort of experimental approach encourage us to evaluate apoptotic related effects in PDT by a direct phase protein microarray platform, namely antibody array, that can offer, in our opinion, a straightforward way for results due to several important advantages such as the known identity of the multiple tested analytes along with a rapid biological interpretation of the data. In addition, the higher versatility of the platform and reproducibility counts for the antibody arrays using in tumor research domain [28]. Reverse phase type of protein microarray is, as much as used in the biomarker discovery, a specific but a laborious type of microarray, with lower standardization and even multiplexing possibilities due to the still low availability for both detection labeled antibodies and specific protein antibodies appropriate for the reverse array platform [29]. In addition, this type of microarrays is prone to cross-reactivity because all the proteins harbored by complex cell cultures are investigated. In our experimental approach, the DOK cell line that we have used has a moderate differentiation stage [30]; therefore the particularities in the apoptotic biomolecules that we came across can be explained by the type of cell differentiation.

## Conclusions

This study is the first one conducted towards elucidation of the molecular mechanisms activated by AlS_2_Pc-PDT in dysplastic keratinocytes using protein microarray analysis. With this high-throughput array technology, traditional methods can be overridden in terms of multiplexing (e.g. ELISA), minimal sample requirements and easy quantification of results. Using this proteomic approach a better understanding of the manner in which photodynamic therapy leads to tumoral cell destruction can be obtained, by depicting apoptotic molecules that can be potentially triggered in future anti-tumoral therapies.

As future perspectives, due to its huge versatility and enormous possibilities of biomedical applications, the protein microarray technology is armed with all benefits for becoming a multiple choice approach for therapy assessing in clinic. Even if the majority of existing arrays platforms are dedicated to fundamental research, namely in biomarkers fields, molecules abundance studies and biomolecules interactions, the number of platforms for clinic purposes is currently increasing. In addition, protein microarray could be an attractive option for both researchers and clinicians because the platform could be customized in terms of number and multiplicity of tested analytes. Molecular changes imprinted by photodynamic treatment in different pathologies are subtle processes and still incompletely revealed, therefore such a fine proteomic technology could explore many uncovered intracellular pathways, contributors to en efficient antitumor host response.

## Methods

### Sensitizer

The procedure of hydroxyaluminum AlS_2_Pc synthesis has been adapted according with some previous literature reports [31, 32], and involved two important steps:

- *Preparation of the sodium salts* of sulphonated hydroxyaluminum phthalocyanine mixture [(OH)AlS_n_Pc], n = 1–4) from hydroxyaluminum phthalocyanine [(OH)AlPc], obtained by the oleum sulphonation of aluminium phthalocyanine, this final product being a complex mixture of mono- to tetrasulphonated derivatives. Briefly, for the sulphonation process an amount of 30 g (OH)AlPc was gradually charged to 300 g of 5% fuming sulfuric acid under constant mixing at 115°C; the reaction mix was kept at 30°C until the (OH)AlPc was fully dissolved. Next, the process of sulphonation was carried out under a nitrogen blanket, with constant agitation and heated up to 120°C for 30 min. After the sulphonation process was completed, the reaction mixture was slowly cooled to room temperature and subsequently charged with a drooping funnel into the vigorously agitated mixture of 2000 g of ice and 1000 g of water. The water suspension was then filtered with a Buchner funnel, the filter cake was washed with distilled water until no sulfate anions were detectable in the filtrate. Thoroughly washed filter cake of sulphonated (OH)AlS_n_Pc was dried at 105°C until a constant weight was attained.
- The contents of sulphonated (OH)AlS_n_Pc were determined by column liquid chromatography (LC), HPLC (high-pressure liquid chromatography) and by thin layer chromatography (TLC) on Baker SI C18 reverse plates. Separation of each sulphonated fraction and the mixtures of sulphonated derivatives, prepared as described above, were performed by LC. In brief, the chromatographic column was filled with silica gel (Kieselgel 60 fur die Saulen, Chrom Merck) and a mixture of ethyl acetate, ethanol and 25% ammonia water (in the volume ratios 7:4:4) was used as a mobile phase. The purity of each dry product was determined by HPLC method, combined with mass spectrometry, which proved the molecular constitution of each separated phase. The purities of the studied (OH)AlS_2_Pc differing in the relative positions of the sulphonated groups, namely *cis* (the groups attached to the vicinal rings) and *trans* (the groups attached to the opposite groups), was assessed. Currently, there is no method of separating the two isomers of (OH)AlS_2_Pc. The purity of each fraction was: (OH)AlS_1_Pc - 97.9 (wt.%), (OH)AlS_2_Pc - 99.4 (wt.%), (OH)AlS_3_Pc - 98.6 (wt.%) and (OH)AlS_4_Pc - 98.2 (wt.%). The spectral characteristics of these components have been recorded by UV–vis spectra at a SPECORD M400 Carl Zeiss Jena spectrophotometer with double beam and microprocessor using MgO as reference solid powder. The following UV–vis λ/ϵ (nm/M^-1^.cm^-1^) values were obtained: (OH)AlS_1_Pc - 678/1x10^5^; (OH)AlS_2_Pc - 1.5x 10^5^; (OH)AlS_3_Pc - 676.6/1x10^5^; (OH)AlS_4_Pc - 676.6/1.6x10^5^.

Stock solutions of AlS2Pc (1.0x10^-5^ M) were routinely prepared in PBS, and stored in the dark at 4°C. For *in vitro* testing, solutions were prepared in culture media and tested concentrations selected on a dose-effect based curve previously explained [5].

### Cell cultures and PDT

Human dysplastic oral keratinocytes, namely standard cell line DOK (ECACC No. 94122104) were cultured in 5% CO2 atmosphere at 37°C in DMEM medium (DMEDM, Sigma-Aldrich, UK) with 2 mM Glutamine, 10% Fetal Calf Serum (Sigma-Aldrich, UK), 5 μg/mL Hydrocortisone (Sigma-Aldrich, UK) and 50U of Penicillin-Streptomycin per 1 mL of cell culture medium (Sigma-Aldrich, UK). Cells were incubated with AlS2Pc at a concentration of 4 μg/ml for 24 h and then irradiated with He-Ne laser source λ = 632.8 nm), following an experimental protocol detailed by the authors in a previous paper [4].

### Apoptosis studies

Cellular apoptosis was verified using Annexin V-FITC (Ann-FITC-green fluorescence) propidium iodide (PI-red fluorescence) kit (BD Biosciences) by flow cytometry. The early feature of apoptosis is characterized by the translocation of the membrane phospholipid phosphatidilserine from internal layer to the external layer of cell membrane, this translocation being identified by high affinity binding of Ann-FITC to phosphatidilserine. In late apoptotic or necrotic cells in which phosphatidilserine translocation has already occurred, the cell membrane is damaged, and cells become double positive for Ann-FITC and PI. Cell samples from the same batches that were subjected to protein microarray analysis were immediately analyzed in a FACScalibur cytometer (Becton Dickinson) within 90 minutes, using Cell Quest software. Results are presented as follows: percentage cells stained double negative – live cells, double positive – dead/necrotic cells and positive for Annexin, negative for propidium iodide – apoptotic cells.

### Protein microarray analysis

Cells incubated with AlS2Pc and control cells, incubated in culture medium were analyzed by protein microarray using *Cancer/Apoptosis Phospho-Specific Array* (Abnova), an analysis kit suitable to detect 155 proteins involved in apoptosis in both their phosphorylated and non-phosphorylated forms. The slides contain antibodies spotted in six replicates for each analyte to be detected, the array being printed on standard-size coated microscope glass. Briefly, after irradiation 1×10^6^ cells from both control and AlS2Pc treated cells were processed according to the kit instructions in order to obtain a cellular lysate which was further purified and labeled with biotin. Prior to coupling reaction and before adding the biotinylated samples, slides were treated for blocking the unspecific binding sites. The coupling reaction and detection with Cy3-Streptavidin were performed afterward and both slides representing control and respectively AlS2Pc treated cells, were scanned using *Full Substrate Spot Light* program (Molecular Devices), with a CCD scanner from *ArrayIt*. Scanned TIFF images of slides were obtained and further analyzed with *Axon GenePix ProV7* software (Molecular Devices), using for calculations the mean values of recorded fluorescence for all spots replicates. After data normalization, results were subjected to statistical normality test with aid of Shapiro-Wilk and Kolmogorov-Smirnov methods, followed by parametric/non-parametric statistical comparison tests (t-test and Mann–Whitney/Wilcoxon tests, respectively).
